# Supplementary material for: The ACE rs1799752 Variant Is Associated with COVID-19 Severity but Is Independent of Serum ACE Activity in Hospitalized and Recovered Patients
Source: Int J Mol Sci. 2023 Apr 21;24(8):7678. doi: 10.3390/ijms24087678 (PMC10142321; doi:10.3390/ijms24087678)
Supplement: Supplementary file 1 [file ijms-24-07678-s001.zip › ijms-2330010-supplementary.pdf]

Supplementary Materials

Table S1. Association study of rs1799752 *ACE* variant with mortality in patients with severe COVID-19.

Allele/Genotype	Non-survivors (n=381)	Survivors (n=742)	p-value [a]
**I**	432 (0.567)	866 (0.584)	0.433
**D**	330 (0.433)	618 (0.416)	
**II**	148 (0.388)	286 (0.385)	
**ID**	137 (0.360)	295 (0.398)	0.304
**DD**	96 (0.252)	161 (0.217)	
**Recessive model**			
**II + ID**	285 (0.748)	581 (0.783)	
**DD**	96 (0.252)	161 (0.217)	0.186
**Dominant model**			
**II**	148 (0.388)	286 (0.385)	
**ID + DD**	233 (0.612)	456 (0.615)	0.922
**Overdominant model**			
**ID**	137 (0.360)	295 (0.398)	
**II + DD**	244 (0.640)	447 (0.602)	0.215

[a] Chi-square test using PLINK v 1.07

Table S2. Study of factor influencing the serum ACE activity in patients with COVID-19.

Continuous Variable	N	Rho value	*p* value*
Age	66	-0.087	0.485
BMI	66	-0.070	0.578
Days since symptoms onset	66	0.037	0.768
**Categorical Variable**		**Serum ACE activity U/L**	***p* value***
Sex			0.345
Male	45	22.5 (13.0-29.9)	
Female	21	22.1 (16.4-33.3)	
Co-morbidities			
T2DM	22	26.2 (14.0-31.4)	0.639
Non-T2DM	44	22.1 (13.9-32.5)	
SAH	22	22.1 (12.9-29.7)	0.924
Non-SAH	44	25.9 (14.1-33.1)	
Heart diseases	3	14.7 (12.7-28.9)	0.723
Non-Heart diseases	63	22.5 (14.0-32.1)	
Respiratory diseases	4	22.3 (21.2-26.8)	0.717
Non-Respiratory diseases	62	23.9 (13.2-32.2)	
rs17991752 *ACE1*			0.165
II	23	22.1 (13.9-27.4)	
ID	22	28.7 (17.2-35.5)	
DD	21	18.6 (10.3-29.9)	

*Mann-Whitney U or Kruskal-Wallis tests, and Spearman correlation test for categorical and continuous variables, respectively. BMI, body mass index; SAH, systemic arterial hypertension; T2DM, type 2 diabetes mellitus

Table S3. Association study of serum ACE activity levels with clinical parameters in patients with severe COVID-19.

Continuous Variable	N	Rho value	*p* value*
PaO_2/FiO_2 at hospital admission	66	-0.039	0.762
IMV days	66	-0.214	0.121
**Categorical Variable**		**Serum ACE activity U/L**	***p* value***
IMV			0.057
IMV	43	19.1 (11.8-29.3)	
Non-IMV	23	28.4 (17.6-49.1)	
Mortality			0.347
Survivor	47	22.1 (14.7-33.8)	
Non-survivor	19	22.0 (11.1-27.4)	

*Mann-Whitney U test and Spearman correlation test for categorical and continuous variables, respectively. IMV, invasive mechanical ventilation.

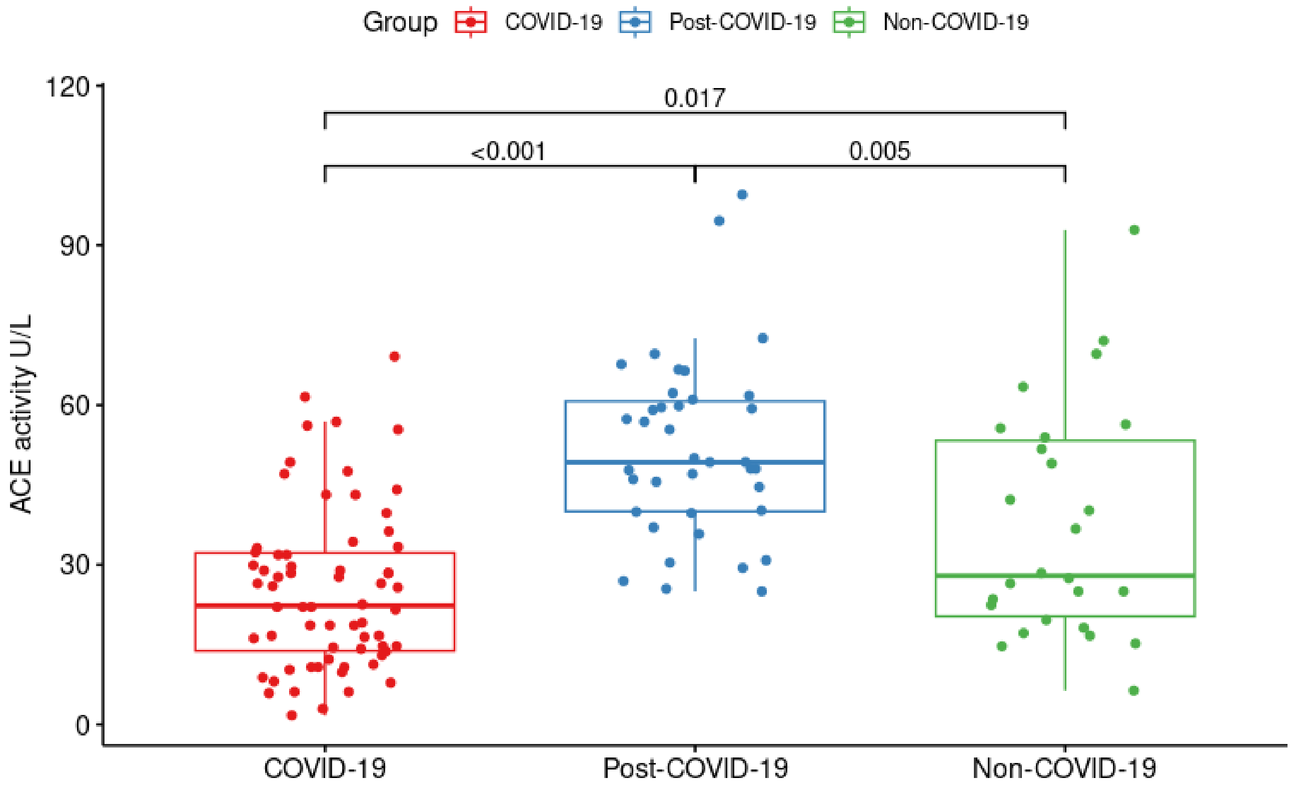

Figure S1. Serum ACE activity levels in the COVID-19 (n=66), post-COVID-19 considered healthy when the sampling was done (n=38), and non-COVID-19 (n=26) groups. The comparisons were performed using Kruskal-Wallis Test corrected with the Benjamini-Hochberg method.
